# Multicenter experiences in temporal bone cancer surgery based on 89 cases

**DOI:** 10.1371/journal.pone.0169399

**Published:** 2017-02-22

**Authors:** Małgorzata Wierzbicka, Kazimierz Niemczyk, Antoni Bruzgielewicz, Marcin Durko, Janusz Klatka, Tomasz Kopeć, Ewa Osuch-Wójcikiewicz, Wioletta Pietruszewska, Marcin Szymański, Witold Szyfter

**Affiliations:** 1 ENT Department, Medical University, Poznań, Poland; 2 ENT Department, Medical University, Warsaw, Poland; 3 ENT Department, Medical University, Łódź, Poland; 4 ENT Department, Medical University, Lublin, Poland; Peking Union Medical College Hospital, CHINA

## Abstract

**Objective:**

To present outcomes of extensive surgery: lateral, subtotal, total petrosectomies in patients with temporal bone invasion resulting from specific primary cancers.

**Study design:**

Retrospective case review.

**Setting:**

Four tertiary referral centers.

**Material:**

89 patients with cancer of the temporal bone treated between January 2006 and December 2010.

**Intervention:**

Multidisciplinary team approach including surgical resection, reconstruction, and postoperative radiotherapy.

**Main outcome measure:**

Disease-specific survival, overall survival.

**Results:**

In 27.0% of the patients, relapse was reported, with an average of 6.3 months after surgery; 31 patients (34.8%) died during the follow-up. The average mortality was 22.1 months. Fifty-four patients (58.7%) stayed alive during the time of observation. The average survival time was 42.0 months. The median time of survival with relapse was 12 months (range: 1–51 months). The three-year disease-free rate was 38.0% and the overall survival rate was 58.7%.

**Conclusions:**

Petrosectomy is an effective treatment for malignant temporal bone invasion. The probability of a good outcome was statistically decreased with a high T grade, positive margins, and salvage surgery. Younger age is connected with better prognosis. One of the major tasks remains to improve detection and to shorten the time to diagnosis, keeping in mind that symptoms are insidious and in younger people, the time before diagnosis was longer.

## Introduction

Squamous cell carcinoma (SCC) of the temporal bone (TB) derived from external auditory canal (EAC) or from the middle ear (ME) is rare, with one to six cases per million annually [1]. Risk factors, including chronic otitis media, past radiotherapy in the neighbouring regions, and exposure to UV radiation (actinic tumours with invasion of EAC) are well documented, but providing evidence-based treatment recommendations is difficult because studies have only reported on small sample sizes [2–4]. To evaluate the survival outcome for ME cancer and to provide patients and clinicians with more accurate estimates of individual survival, probability prognostic models were constructed [5]. Skin cancers and parotid cancers that have invaded the temporal bone are over ten times more frequent, but these cases have even less evidence on best practices [6].

Regardless of the origin of the primary cancer, combined surgery and radiotherapy remains the treatment of choice for the majority of these malignancies [7]. However, the effectiveness of preoperative chemoradiotherapy [8], of arterial chemotherapy [9], of the employment of carbon ion radiotherapy [10], and of radical radiation therapy (RT), comprising intracavitary brachytherapy as a boost after external beam RT [11], suggests new perspectives are needed in TB cancer treatment. Though there are advances in modern imaging and irradiation, this rare and difficult clinical entity is still a challenge for the surgeons, thus only a finite number of departments deal with this type of surgery. To achieve results, collaboration between the referral centres is needed [12,13]. The aim of this study was to present the outcomes of patients with TB invasion who underwent petrosectomies in four university departments, with special regard to survival in advanced stages of the disease, and with particular emphasis on the primary tumour location and its impact for treatment feasibility and outcomes.

## Materials and methods

To address the research purpose, the investigators designed and implemented a retrospective analysis of 89 patients (39 males—43.8%, 50 females—56.2%) who had lateral skull-based tumours and were referred to the university departments of otolaryngology at tertiary referral centres in Poznan, Warsaw, Lodz, and Lublin for surgery between January 2006 and December 2010. All patients were operated on with the intention to cure. The mean age of patients was 64.8 ±15 years 95%CI (59.9; 69.6). The histology was predominantly squamous cell carcinoma (SCC). The patients’ details are shown in Table 1.

**Table 1:** Characteristics of patients with temporal bone malignancies from four University Departments.

	No / percentage of patients
**Precise tumor localization**	
1. Pinna/ external ear	8 (9.0%)
2. External auditory meatus (EAM)/ Middle ear (ME)/Inner ear (IE)	68 (76.4%)
3. Parotid gland	13 (14,6%)
**Tumor extension**	
Skin involvement	53(59.6%)
Tumor adjacent/infiltrating the dura (D)	33(37.1%)
Temporo-mandibular-joint (TMJ)	24(27.0%)
**TNM**	
T1	1(1.1%)
T2	7(7.9%)
T3	5(56%)
T4	76(85.4%)
N0	63(70,8%)
N1	24(27.0%)
N2	2(2,2%)
**Previous treatment**	
Surgery	38 (42.7%)
RT	19 (21.3%)
**Histopathology**	
squamous cell carcinoma (SCC)	64 (71.9%)
adenoid cystic carcinoma (ACC)	13(14.6%)
skin cancers	9 (10.1%)
malignant melanoma (MM)	3 (3.4%)

The revised Pittsburgh system for the EAM was used to classify TB and lateral skull-based tumours [14,15] The American Joint Committee on Cancer (AJCC) TNM staging system was used for parotid tumours [16]. The progression of the primary tumour was assessed using computerized tomography (CT) and magnetic resonance imaging (MRI). A preoperative biopsy was performed on all patients. For four patients with false negative biopsies, frozen sections were taken during the surgical procedure. The stage of lymph node involvement (N) was assessed based on a neck CT, an MRI, and an ultrasound (US). Audiological assessments were performed preoperatively for all patients. Seventy-six patients had T4 tumours, twenty-six had neck metastases, and four (4.4%) had no data available. None of the patients had distant metastases. The detailed tumour advancements and their extension to the neighbouring anatomical structures are presented in Table 1. The patients’ follow-up visits occurred between 36 and 60 months (median 51 months) after their surgeries. The statistical analysis was performed by SPSS software (Version 10). The overall survival (OS) rates were calculated by the Kaplan-Meier method. Differences between the groups were estimated using the log-rank test. Multivariate analyses were performed using the Cox regression model. A probability level of 0.05 was chosen for statistical significance.

The main predictor variables studied were the T stage and the origin of the primary tumour (external ear versus EAC/ME/internal ear(IE) versus parotid), the type of surgery, the presence of free margins, the N stage were additional predictor variables. Primary outcome variables studied were the following: the recurrence rate, a three-year disease-free rate, and the overall survival rate. Age, sex, duration of disease history, FN paresis at presentation, and pathological types also are variables that could be related to the outcomes. The Kaplan-Meier test was used to analyse disease-specific and overall survival rates for the entire cohort.

### Compliance with ethical standards

#### Conflict of interest

The authors declare that they have no conflict of interest.

All procedures performed in studies involving human participants were in accordance with the ethical standards of the institutional and/or national research committee and with the 1964 Helsinki declaration and its later amendments or comparable ethical standards. For this type of study formal consent is not required.

This article does not contain any studies with animals performed by any of the authors.

Our study was approved by Bioethical Commission.

Patients records were de-identified and analyzed anonymously.

## Results

The mean age of the female participants was 58.6 years (median = 58.0, range = 20–87 years old; 75% were under the age of 70). The mean age of male participants was 64.8 years (median = 67.0, range = 29–89 years old; 75% were under the age of 76). There was significant (6 years) difference in age between the male and female participants (p = 0.03734, Mann-Whitney U Test). Characteristics of patients with temporal bone malignancies is presented in Table 1.

The most common first symptom was ear discharge observed in 39 patients (43.8%: 13 male, 26 female), and then pain, observed in 38 (42.7%: 11 males, 27 females), followed by an equal number of patients (24: 26.9%) with lump or ulceration (16 males, 8 females), and hearing impairment (8 males, 16 females). Facial total palsy was the first sign of disease in 15 patients (16.9%: 10 males, 5 females). Other complaints were observed in 5 patients (5.6%). The facial nerve (FN) status in the House-Brackmann scale (HB) was assessed at presentation and was as follows: grade 6: 15 (16.9%), 5: 13 (14.6%), and 4: 7 (7.9%).

### Time before diagnosis

The mean time before diagnosis was 2.2 ± 2.5 years 95%CI (1.6; 2.6). The median was 1 year. For males, 1.6 years (median = 1.0 year; range = 0.5 months to 6 years). 75% of the women were diagnosed before 3 years, with time ranging from 0.5 months to 14 years. 75% of the males were diagnosed in less than 2.5 years. However, these figures do not represent a significant difference between males and females. There was a weak correlation between age and time before diagnosis (Spearman coefficient of correlation r = -0.21); younger patients were diagnosed later than were older patients. There was no relationship between the T stage and gender or age or between the N stage and gender or age.

### Localization of the primary

The predominant localization of the primary cancer (76.4% patients) was EAC/ME. There was no difference in particular localization of the primary cancer and the patients’ age (H (2; N = 89) = 3.397705 p = 0.1829) and gender(Chi2(2) = 1,4, p = 0.50770). There was, however, a significant difference between localization and histology (Pearson Chi-square p = 0.00001). There was no significant difference in time before diagnosis and primary localization (H (2; N = 89) = 3.397705 p = 0.1829), Kruskal-Wallis ANOVA by Ranks). The mean for time before diagnosis in patients with cancer in the external ear was 3.7 years (median = 2 years). There was no difference between localization and previous treatment, T grade or lymph node involvement. There was no significant relationship between the localization and the FN paresis at presentation (Pearson Chi-square p = 0.06004); 69.23% of patients with a parotid gland had the paresis, whereas 83.3% and 60.9. % of patients with external ear cancer and EAM, tympanic cavicty (TC), or IE had no paresis. The comparisons between primary localizations and localisation and histology are presented in Tables 2 and 3.

**Table 2:** Precise localization and histology.

	Histology				
	SCC	MM	ACC	Skin cancers	Total
**Precise localization**					
Pinna,external ear	8	0	0	0	8
EAC,TC,IE	51	3	5	9	68
Parotid gland	4	0	8	0	12
Total	63	3	13	9	88
%	71,59%	3,41%	14,77%	10,23%	100,00%

**Table 3:** Comparison between the primary localizations.

	Localization of the tumour			Statistic parameters
	Pinna, external ear	EAM, ME, IE	Deep parotid lobe	
**Time to diagnosis (years)**	3.7	1.6	3.9	Kruskal-Wallis: H (2, N = 89) = 6,626050, p = 0.0364
**Histology**	-	SCC	ACC	Chi2(6) = 32,58627 p = 0.00001
**FN paresis at presentation**	12,5%	25,3%	69,3%	Chi2(2) = 10.22107 p = 0.00603
**Treatment: parotidectomy**	75%	35,2%	100%	Chi2(2) = 14.38219 p = 0.00075
**FN intentional resection**	25.0%	29.4%	69,2%	Chi2(4) = 6.260435 p = 0.04371
**TMJ resection**	25.0%	16.2%	69.2%	Chi2(2) = 16.59020 p = 0.01091
**Positive margins**	20.6%	23.1%	25,0%	Chi2(4) = 13.19099 p = 0.15415

### Type of surgery (Table 4)

Total petrosectomy (TTBR), subtotal petrosectomy (STBR), and lateral petrosectomy (LTBR) were performed in 14, 28, and 47 patients, respectively. In patients with total petrosectomy resection of a portion of the petrous bone including inner ear and petrous apex was performed. There was no significant relationship between precise localization and type of surgery (Chi2 (2) = 0.5494506; p = 0.75978). Total parotidectomy was performed in all patients with parotid gland malignancies, in 75% and 35.2% of primaries in the external ear and EAC, TC, or IE, respectively (Chi2(2) = 14.38219 p = 0.00075). Resections of the FN in 25, temporomandibular joint (TMJ) in 22, and dura in 27 patients were performed. The intraoperative findings showed that there was no relationship between primary localization and involvement of the FN, the skin, or the dura. There was, however, a significant relationship between the primary localization in the parotid (64.7% of patients) and in the TMJ invasion (Chi2(2) = 9.347663 p = 0.00934).

**Table 4:** Details of the surgical treatment and outcome.

	No / percentage of patients
**Type of surgery**	
Total petrosectomy	14 (15.7%)
Subtotal petrosectomy	28(31.5%)
Lateral petrosectomy	47(52.8%)
**Parotidectomy**	
Lateral	10 (11.2%)
total	44 (49.4%)
**Resection of adjacent structures**	
Intentional excision of the FN	25(28.1%)
Peeling /resection of the dura	23/5; 28 (31.5%)
TMJ	22(24.7%)
**Positive margins**	19 (21.3%)
**Additional treatment (adjuvant RT)**	46 (51.7%)
**Survival rate**	
T1, T2, T3	100%
T4	51,3%

There was a significant relationship between the primary localization and the FN intentional resection (Chi2(2) = 6.260435 p = 0.04371); it was required in 69.23%, 12.5%, and 21.1% of patients with parotid malignancies, external ear, and EAC, respectively. There was also a significant relationship between primary localization and TMJ resection (Chi2 (2) = 11,71875 p = 0.00285); it was required in 72.7%, 28.6%, and 25.5% of patients with parotid, external ear, and EAM, respectively. There was no statistically significant relationship between localization and the need of a dura resection.

The margins were positive in 19 patients. There was no significant relationship between the localization of the primary cancer and the positive margins. Additional treatment was required in 51.7% of the patients. There was no significant relationship between the application of the additional treatment and the primary localization, nor with age.

### Reconstruction

Large skin defects were covered with microvascularized anterolateral tight (ALT) - 9 and forearm radial flaps (FR) - 5. Smaller defects were closed by simple skin advancement, rotation, or transposition flaps– 48. Reconstruction of the dura was performed in five patients. Small defects were managed using a collagen sponge with human fibrin using a “sandwich” technique. Larger defects were covered by fascia. In four patients (4.5%), an FN cable graft from the middle ear to the intraparotid branches using the greater auricular nerve was performed. Other patients underwent a static procedure, an oral commissure cheek suspension with Goretex,® three months after radiotherapy.

### Oncological outcomes

We analysed different patient-related, tumour-related, and treatment-related factors that could possibly have an impact on survival. In 27.0% of the patients, relapse was reported, with an average of 6.3 months after surgery; 31 patients (34.8%) died during the follow-up. The average mortality was 22.1 months. Fifty-four patients (58.7%) stayed alive during the time of observation. The average survival time was 42.0 months. The median time of survival with relapse was 12 months (range: 1–51 months). The three-year disease-free rate was 38.0% and the overall survival rate was 58.7%.

**Fig 1:**
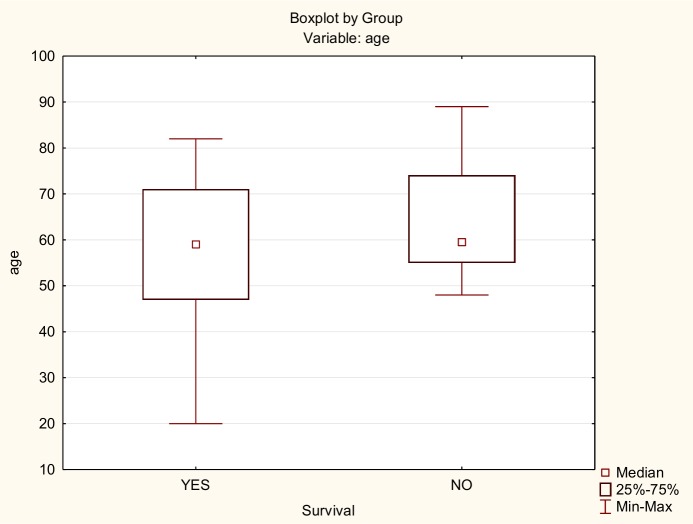
Difference in age between alive and death patients.

**Fig 2:**
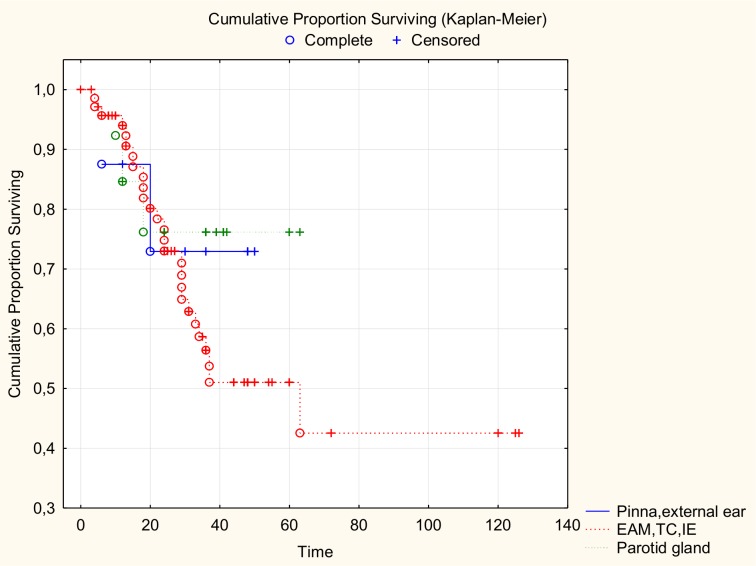
Survival analysis for the group of 89 patients with temporal bone invasion, considered due to localization of the primary.

**Fig 3:**
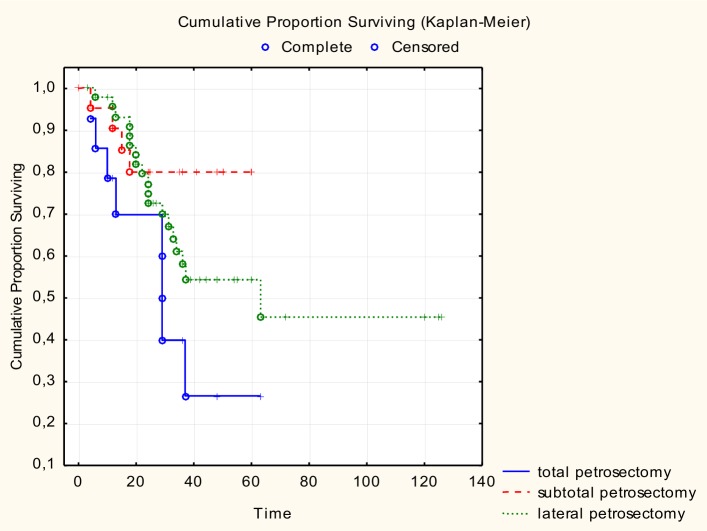
Extent of the surgery and survival.

There was no significant difference in age between the patients who lived and those who died, although the younger patients did better (Fig 1). There was no significant relationship between tumour localization and survival; the survival curves for the three groups were similar (Fig 2). Patients with T1, T2, and T3 tumours had 100% survival. Patients with stage T4 had a survival rate of 51.3%. There was a significant relationship between survival and tumour grade (Chi2 (3) = 10.83224; p = 0.01267), previous treatment (Chi2 (1) = p = 0. 04871), and positive margins (Pearson Chi2 (1) = 3,528510; p = 0.04070): 63.2% of the patients with positive margins died, and 73.33% patients with negative margins stayed alive during the follow-up. There was no statistically significant difference in survival due to the extension of surgery (Chi2(2) = 4.57; p = 0.10158) (Fig 3).

Patients, except those who had been previously irradiated, received RT at a total dose of 64-72Gy, six weeks after surgery. There was no significant relationship between survival and adjuvant radiotherapy in group, which was irradiated after surgery.

Eleven patients suffered postoperative complications, including: myocardial infarction, aspiration resulting in bronchopneumonia, and stroke with hemiplegia. Patients suffered infections and dehiscence of the wound necessitating secondary sutures. Three of the free flaps required revision of the anastomosis, but none of the flaps were lost.

## Discussion

We presented the data from surgeries on 89 patients with TB malignancies from four tertiary referral centres, covering the majority of a population of 40 million. The unique value of the reported sample is the size, which was a relatively homogenous group, having a high advancement of the disease (T4-82.6%), and having had longer than 36-months of observations for the whole sample.

Late diagnosis is typical for TB tumours. The diagnosis is often delayed because the disease may be masked by other ear symptoms. The classic triad of symptoms consists of discharge, pain, and bleeding present for more than 12 months at diagnosis [7]. In our series, the most common symptoms are those mentioned above, but the time before diagnosis was even longer (2.2 years). The reasons behind the prolonged diagnosis seem to be: unawareness of the symptoms by the patient and the attending physician. There were also significant differences between symptom duration and primary localization categorized as follows: pinna/external ear (3.7 years), EAC/ME/IE (1.6 years), and parotid (3.9 years). The noticeable differences were caused by delayed presentation for surgery in four patients with skin cancers originating in the pinna region. The even longer delay (7 and 9 years) occurred in two patients with *carcinoma ex pleomorphae adenoma*. Somewhat surprisingly, the presence of FN paresis did not reduce the time before hospital admission. An important finding was that young age negatively influences the time before diagnosis.

Combined surgery and radiotherapy is the treatment of choice in all temporal bone tumours. The complex anatomy of the temporal bone, and the delayed presentation and diagnosis make the surgical management difficult. Surgical resection options include LTBR or extended temporal bone resection (ETBR) [17]. Some authors, in addition, have distinguished between lateral, subtotal, and total petrosectomies [18]. The only factor that affected the recommended treatment was the extent of the tumour [19,20]. For advanced cases, *en bloc* or piece meal resection with tumour-free surgical margins remains the unquestioned oncological principle [21,22]. LTBR is advocated with smaller tumours [17]. Parasad and Janecka reviewed English language reports on TB SCC and concluded that patients with disease extending into the ME were more likely to survive for five years with STBR (42%) than were those with LTBR (29%) or mastoidectomy (17%) [22]. Survival rates were negatively influenced by an invasion of the internal carotid artery or a jugular foramen, neck metastases, lack of free margins, and poorly differentiated histology [19,23,24]^.^ Where the dura, the brain parenchyma, or the internal carotid artery was involved, resection of these areas did not appear to improve survival [1,21]. Some authors reported positive results for excision of the internal carotid artery with a saphenous vein graft reconstruction, but later reports provided no evidence that more extended resection was beneficial [23–25]. In our series, the extent and type of surgery, as well as a dura resection and reconstruction had no impact on survival. The internal carotid artery had not been infiltrated and sacrificed in any of our cases.

Positive margins are an indication of postoperative radiotherapy. Although the effectiveness of adjuvant RT remains controversial, some studies have demonstrated an improvement in terms of the survival rate and local control compared with patients who only underwent surgery [26,27]. However, other authors concluded that a positive surgical margin was the major cause of recurrence, and adjuvant radiotherapy showed no more effect on survival [28]. In the presented series, positive margins were a very strong negative factor correlating with poor survival, and the adjuvant RT did not improve the prognosis.

The majority of recurrences are reported to develop within 12 months of initiating treatment [29]. In our series, a 27.0% rate of relapse was reported, all of which occurred in T4 cases, with a mean time until recurrence of 6.4 months. The median survival time after relapse was 8 months. Although patients with primary parotid cancer and non-T4 primary temporal bone cancer did better, the differences were not significant, which was probably due to the unequal numbers in our groups.

Evaluation of the prognosis of middle ear cancer is challenging because of its rarity. Prognostic models to predict overall and cause-specific survival for patients with primary ME cancer, based on an analysis of 247 patients demonstrated good accuracy in predicting survival [5]. In a multivariable analysis, age, histological subtype, stage, surgery, and radiotherapy were predictive of survival. Prognosis was worse with increasing age, SCC histology, and distant disease than it was for those with a localized and regional disease [5]. However, subsets of patients, such as those with adenocarcinomas and with localized tumours, demonstrated significantly better survival rates. Prognosis for T3 and T4 disease is much worse; reported five-year survival rates vary from 14% to73% [24]. In the presented series, the survival was significantly influenced by the T stage, salvage surgery, and positive margins. We confirmed that prognosis in temporal bone SCC is good in early, T1, and T2 tumours [1,30]. Younger age was associated with a better outcome, but not to the point of significance.

The prevalence of lymph node involvement in temporal bone cancers at presentation varies from 10% to 23% but is connected with a grave prognosis [18,24]. Some authors have routinely recommended elective neck dissection for these cases and the UK Ear Nose and Throat guidelines suggested this procedure [6,24]. Among our participating patients, neck metastases reached 30% but did not worsen the prognosis. Reconstruction of skin defects with local or free flaps was indispensible [31–33]. In our group, there were apparent differences between the departments in the reconstruction types. Although the free flaps are clearly better for aesthetic reasons, more reliable in irradiated fields, and better where postoperative RT is planned, they were not achievable in all four departments.

Our study has numerous potential limitations. The retrospective character prevented us from obtaining certain clinical data. Using multiple centres for patients’ collection resulted in some differences in the archives, and these have remained despite the arrangements. However, collaboration between departments dealing with this type of surgery is needed to reinforce the results. The next issue is, that most of the research teams were committed to the homogeneity of their studies [4,5,8,11,12,34]. Our group was not uniform as to history of previous treatment, histology, or implementation of the adjuvant treatment. On the contrary, one of the goals of this study was to compare the different primary tumour localizations with TB invasion as a common denominator. On the one hand, such a design of the study weakens the strengths of inference, but on the other, allows for a comparison of the differences between particular primaries as to the time before diagnosis, histology, FN paresis, and its intentional resection. The important conclusion from this analysis is the lack of differences in survival between particular primaries, distinct histology, and application of postoperative radiotherapy.

In summary, our findings elucidate certain aspects of the TB malignancies. Combined petrosectomy and radiotherapy is an effective treatment for malignant TB invasion. The probability of a good outcome was statistically decreased with a high T grade, positive margins, and salvage surgery. Younger age is connected with better prognosis. One of the major tasks remains to improve detection and to shorten the time to diagnosis, keeping in mind that symptoms are insidious and in younger people, the time before diagnosis was longer.
